# Decoding Lung Cancer at Single-Cell Level

**DOI:** 10.3389/fimmu.2022.883758

**Published:** 2022-05-23

**Authors:** Xing-Xing Fan, Qiang Wu

**Affiliations:** ^1^Dr. Neher’s Biophysics Laboratory for Innovative Drug Discovery, The State Key Laboratory of Quality Research in Chinese Medicine, Macau University of Science and Technology, Macau, China; ^2^The State Key Laboratory of Quality Research in Chinese Medicine, Macau University of Science and Technology, Macau, China

**Keywords:** lung cancer, single-cell analysis, scRNA-seq, tumor microenvironment, drug resistance

## Abstract

Lung cancer is the leading cause of cancer death due to its high degree of malignancy, rapid growth, and early metastasis. Recent studies have found that lung cancer has a high degree of heterogeneity which is characterized by the mixture of different tumor cell types. However, the driving genetic/epigenetic mechanism of lung cancer heterogeneity, how different types of cells interact, and the relationship between heterogeneity and drug resistance have been poorly understood. Single-cell technology can decompose high throughput sequencing information into each cell and provide single-cell information in high resolution. By using single-cell analysis, researchers can not only fully understand the molecular characteristics of different cell types in the same tissue, but also define completely new cell types. Thus, single-cell analysis has been widely utilized in systems biology, drug discovery, disease diagnosis and precision medicine. We review recent exploration of the mechanism of heterogeneity, tumor microenvironment and drug resistance in lung cancer by using single-cell analysis. We propose that the recent findings may pave new ways for the treatment strategies of lung cancer.

## 1 Introduction

As the most common cause of cancer mortality, lung cancer results in millions of death every year in worldwide (1). Among numerous mechanisms underlying lung cancer progression, tumor heterogeneity is the main cause for drug resistance and tumor relapse. The bulk of tumor tissue consists of a diversity of tumor cells possessing distinct genetic signatures which are closely related to their sensitivity to anti-cancer treatments (2). Therefore, the features of tumor cells are dynamically changed when treated with different therapeutic strategies. This enables lung cancer to adapt and further resist to chemical-, targeted- and immune-therapies.

Intratumoral heterogeneity includes both cellular and non-cellular components in tumor microenvironment (TME) and can facilitate tumor initiation and metastasis (3). It is a critical factor which directly correlates with the clinical responsiveness to cancer therapy, and is also associated with the TME which characterized by different abundance and types of cancer stem cells, immune and stromal cells (4). Among these TME cell types, cancer stem cells are responsible for the diversity of tumor cells, while the frequency of tumor infiltrating lymphocytes determines the clinical efficiency of immunotherapy (5) and stromal cells function as the backbone to support tumorigenesis (6).

Therefore, utilizing advanced technologies to systematically analyze personalized cancer mutation and TME will provide meaningful insights for understanding biological foundations and designing novel anti-tumor strategies. In this minireview, we summarized most recent applications of single-cell analysis in lung cancer research and therapies.

## 2 Single-Cell Analysis

Single-cell analysis refers to comprehensive studies of the genome, transcriptome, and proteomics to identify the genetic material and RNA or protein profiling differences between cells with high resolution to better understand the microenvironment of a single cell (7). Since single-cell RNA sequencing (scRNA-seq) was firstly reported in 2009 (8), Single-cell analysis has been extensively further developed and integrated with other technology, including epigenetic sequencing, proteomics and spatial transcriptome sequencing (9). These advanced technologies have significantly enhanced the ability of data acquisition and thus single-cell analysis has been widely used in many fields.

As a rapidly developed technology, scRNA-seq enables researchers to reveal the functions and characteristics of cells at different stages and different locations (7). It has been widely used to compare the differential expression of genes in thousands of single cells and explore drug action mechanism and specific disease associated pathways (10). Furthermore, single-cell proteomics has been developed to explore mechanisms of how dynamics of proteome and cellular phenotypes are regulated though ultra-high sensitivity and high-resolution biological mass spectrometer are required to analyze limited amount of protein samples (11). Recently, combination of different technologies with single-cell analysis allows researchers to identify how gene-regulatory network determines cell fates. For example, Yan et al. developed a single-cell multi-omics technology (scNOMeRe-seq) which can investigate chromatin accessibility, DNA methylation, and RNA expression in parallel in the same individual cell with high accuracy, sensitivity, and genome coverage. By using scNOMeRe-seq, the researchers were able to depicted genetic and epigenetic regulatory landscape at single-cell resolution in mouse early embryo (12).

## 3 Applications of Single-Cell Analysis in Lung Cancer Research

Lung cancer is the most common cause of cancer related death, with less than 21% five-year survival rate (1) which is mainly due to lack of treatment options. To enhance the clinical efficacy, identification of new targets and establishing novel therapeutic strategies are required. As a highly-effective and reliable tool for human cancer research, single-cell analysis has been extensively used to discover tumoral heterogeneity, characterize surrounding microenvironment, and study tumor response towards clinical therapies (13).

### 3.1 Revealing Tumor Heterogeneity of Lung Cancer

Single-cell analysis provides a superior way to assess and measure profiling of RNA or protein pool from numerous tumor cells and subsequently discover new targets for cancer therapy and overcome drug resistance. The molecular subtypes of lung cancer can be clearly defined and novel cancer driver genes can be identified by single-cell analysis. Subsequently, the pathological mechanisms of lung cancer can be further explored, leading to the development of clinical treatments. As lung cancer could mainly be divided into two subtypes: non-small cell lung cancer (NSCLC) and small cell lung cancer (SCLC), we will discuss them separately as following.

#### 3.1.1 NSCLC

To analyze the heterogeneity of NSCLC, Maynard et al. utilized scRNA-seq to examine a large amount of lung cancer samples at different time points of cancer treatment (14). Interestingly, these cells displayed an extremely complex and dynamic tumor ecosystem. Cancer cells that survived from treatments exhibited a signature of alveolar cell pathway activation, suggesting a transition to a primitive cell state in cancer cells (14). This implies that cancer cells and the TME become more plastic upon anticancer drugs. In addition, Wu et al. analyzed the biopsy samples of 42 NSCLC patients and provided a very comprehensive map of TME and heterogeneity for advanced lung cancer samples. The authors demonstrated that tumors from different patient sources have significant heterogeneity in cellular composition, chromosomal structure, differentiation trajectories, and intercellular signaling networks (15). The results from this single-cell analysis could provide more accurate clinical diagnosis and prognosis prediction and facilitate new anti-lung cancer drug development.

#### 3.1.2 SCLC

How the heterogeneity of SCLC is established and different types of cells interact with each other has remained unclear for a long time. Recently, Wu et al. utilized scRNAseq coupled with immunohistochemistry to investigate lung neuroendocrine cells and successfully identified YAP/TAZ signally as a critical component of tumor heterogeneity and drug resistance in SCLC. They found that activating YAP in SCLC cells significantly inhibited the expression of neuroendocrine-like genes in SCLC and promoted the differentiation of tumor cells. The researchers also found that YAP-activated tumor cells developed significant resistance to the chemotherapy drugs Cisplatin and Etoposide (16). This study revealed the regulatory mechanism of YAP/TAZ signaling pathway in the establishment of intratumor heterogeneity and drug resistance in SCLC. Hence, YAP/TAZ may be a novel target for SCLC therapy.

By generating circulating tumor cell (CTC)-derived xenografts (CDXs) from SCLC patients and utilizing scRNA-seq, Stewart et al., 2020 investigated the intratumoral heterogeneity of SCLC cells. They revealed that heterogeneous expression of therapeutic targets and potential resistance pathways were globally increased. Furthermore, chemosensitivity related markers were rapidly downregulated and various new subsets of SCLC cells emerged in post-treatment, resulting in the diversity of tumor cells and occurrence of resistance (17). Lu et al. used scRNA-seq to identify 8 types of cells in TME, including cancer cells, endothelial cells, fibroblasts, T cells, B cells, Nature killer cells, mast cells, and myeloid cells. This provided a comprehensive description of cell phenotypes in ground glass nodules adenocarcinoma (GGN-ADC). can shed new lights into underlying mechanisms of lung cancer carcinogenesis including signal pathway and T cell activation (18).

To investigate the heterogeneity of SCLC tumors and the associated microenvironments, Chan et al. massively carried out single-cell sequencing and observed greater tumor diversity in SCLC than lung adenocarcinoma. The authors also identified a PLCG2-high, stem-like SCLC population which was speculated to be associated with immunosuppressive and recurrent SCLC tumors (19).

In sum, single-cell analysis has remarkably uncovered the heterogeneity and TME of lung cancer with high resolution. These new findings can promote the novel drug target discovery and precision therapeutic strategies against lung cancer.

### 3.2 Defining TME

The TME is a complex ecosystem in which cancer cells, immune cells, stromal cells, and their interactions constitute a fine regulatory network that determines the occurrence and development of cancer. The status of TME is directly related to the efficiency of immunotherapy (20). Thus, the immune contexture in TME, including the composition, enrichment, and functional state of infiltrating lymphocytes, possesses the predictive information relevant to prognosis and treatment response. Single-cell analysis can determine the local immune contexture and facilitate the discovery of immunological biomarkers of oncology.

As stroma is composed of extracellular matrix and supports tumor cells by providing nutrition and removing waste products, Lambrechts et al. identified various stromal cell subgroups and found dynamic changes in gene expression in lung carcinoma. Therefore, the unique features of the tumor stroma may be an entry point for novel therapeutics (21). Besides, Lavin et al. used scRNA-seq to analyze the tumor immune microenvironment of 18 lung adenocarcinoma patients and found the significant reduction of CD8+ T cells expressing granzyme B in lung cancer cells compared with normal lung cells. This study also demonstrated that depletion of CD141+ dendritic cells and enrichment of PPARγhi macrophages, and reduced NK cells in early stage lung cancer can be potentially used as biomarkers for cancer therapy (22).. Another study analyzed the single-cell sequencing data of NSCLC patients and found that a high “pre-exhausted” to exhausted T cells was associated with better prognosis of lung adenocarcinoma (23). At the same time, they also observed heterogeneity within tumor regulatory T cells (Tregs) was associated with poor prognosis in lung adenocarcinoma (23). Zilionis et al. further localized tumor-infiltrating myeloid cells in patients with NSCLC and suggested that tumor-infiltrating myeloid cells may become new targets for immunotherapy (24). Chen et al. have revealed that the diversity of B cells in NSCLC is critical for anti-tumor therapy (25). The presence of M1-like macrophages marked by CXCL and NF-κB in lung cancer, was associated with inflamed tumor and good prognosis (26). Single-cell analysis is also important for studying T cell heterogeneity and understanding their activation, differentiation, and regulatory mechanism within TME. Li et al. established a computational method to comprehensively detect and annotate isoforms on a scRNA-seq data and successfully typed tumor-infiltrating T cells (27). Taken together, single-cell analysis has highlighted the crucial regulatory roles of immune cells in lung cancer and can clearly decode the complexity of TME.

### 3.3 Unraveling Mechanisms of Lung Cancer Invasion and Metastasis

The malignancy of lung cancer is largely due to invasion and metastasis which involves a series of steps gene expression pattern changes. It remains a key issue to address how underlying mechanisms drive some lung cancer cells to become metastatic. More and more evidences support that TME plays a fundamental role in regulating the behavior of cancer cells and TME components mediate cancer cells to become invasive and disseminate from the primary site to distant locations (28). Therefore, decoding TME maintains critical to unravel the mechanism of cancer metastasis. Kim et al. investigated single-cell transcriptome profiling of metastatic lung adenocarcinoma. Cancer cell subtypes that deviate from normal differentiation trajectories and dominate metastasis were identified. At all stages, dynamics of stromal and immune cells created a tumor-promoting and immunosuppressive microenvironment (29).

Circulating tumor cells (CTCs), as the actively metastasizing cells, are the main targets of liquid biopsy and important biomarkers for tumor prognosis monitoring (30). The study of CTCs in lung cancer patients based on single-cell sequencing technology can not only discover new CTC biomarkers, but also reveal the metastasis mechanism of tumor cells at the molecular level. Ni et al. observed characteristic cancer-associated single-nucleotide variants in the CTC exome by whole-genome sequencing and found a high degree of consistency in copy number variation (CNV), indicating that CNV may be associated with tumor metastasis. This observation may provide a basis for future CNV-based lung cancer diagnosis and treatment (31). In addition, Su et al. monitored somatic mutations and copy number alteration (CNA) by single-cell sequencing of CTCs from SCLC patients during chemotherapy. The results of this study showed that patients with low CNA scores had significantly longer progression-free survival and overall survival after first-line chemotherapy compared with high CNA scores (32).

### 3.4 Uncovering the Underlying Mechanism of Drug Resistance

Although the advanced lung cancer therapy has made great progress, especially the development and application of immune checkpoint inhibitors, how to explore the drug resistance mechanism and develop more effective prognostic markers has largely remained unknown. Utilizing single-cell analysis to compare the primary and drug resistant cancer cells could shed new light on this line of research. Kim et al. isolated patient-derived xenograft (PDX) lung tumor cells and performed single cell RNA-seq for gene expression profiling and expressed mutation profiling. They observed heterogeneous tumor-specific single-nucleotide variations, including KRAS G12D in individual PDX cells. Interestingly, PDX cells that survived *in vitro* anti-cancer drug treatment, displayed transcriptome signatures consistent with the group characterized by KRAS G12D and low risk score (33). Thus, single-cell analysis can be used to identify drug resistance-related signature genes. Moreover, single-cell analysis can dynamically monitor the shift from a pre-existing immune response to a therapy-induced immune response and thus provide evidence for further therapy. This is critical for exploring resistant mechanisms to current therapy, and thus develops overcoming and synergistic strategies to enhance the clinical efficacy (34).

Although tumor mutation burden (TMB) is associated with response to immunotherapy which is the main therapy of NSCLC, little has been known about the relationship between baseline immune response and tumor genotype. Recently, a comprehensive single-cell RNA analysis of early-stage NSCLC cells identified a cellular module consisting of PDCD1^+^CXCL13^+^ activated T cells, IgG^+^ plasma cells, and SPP1^+^ macrophages, termed the lung cancer activation module (LCAM^hi^). The researchers confirmed the enrichment of LCAM^hi^ in multiple NSCLC cohorts and established an antibody panel to identify LCAMhi lesions by paired CITE-seq. The researchers found that the presence of LCAM was independent of overall immune cell content and correlated with TMB and high baseline LCAM scores were associated with enhanced response to immunotherapy in NSCLC (35). Therefore, this study has shown that the TMB-correlated immune cell composition could be a potential biomarker of response to immunotherapy.

## 4 Perspectives

**Figure 1:**
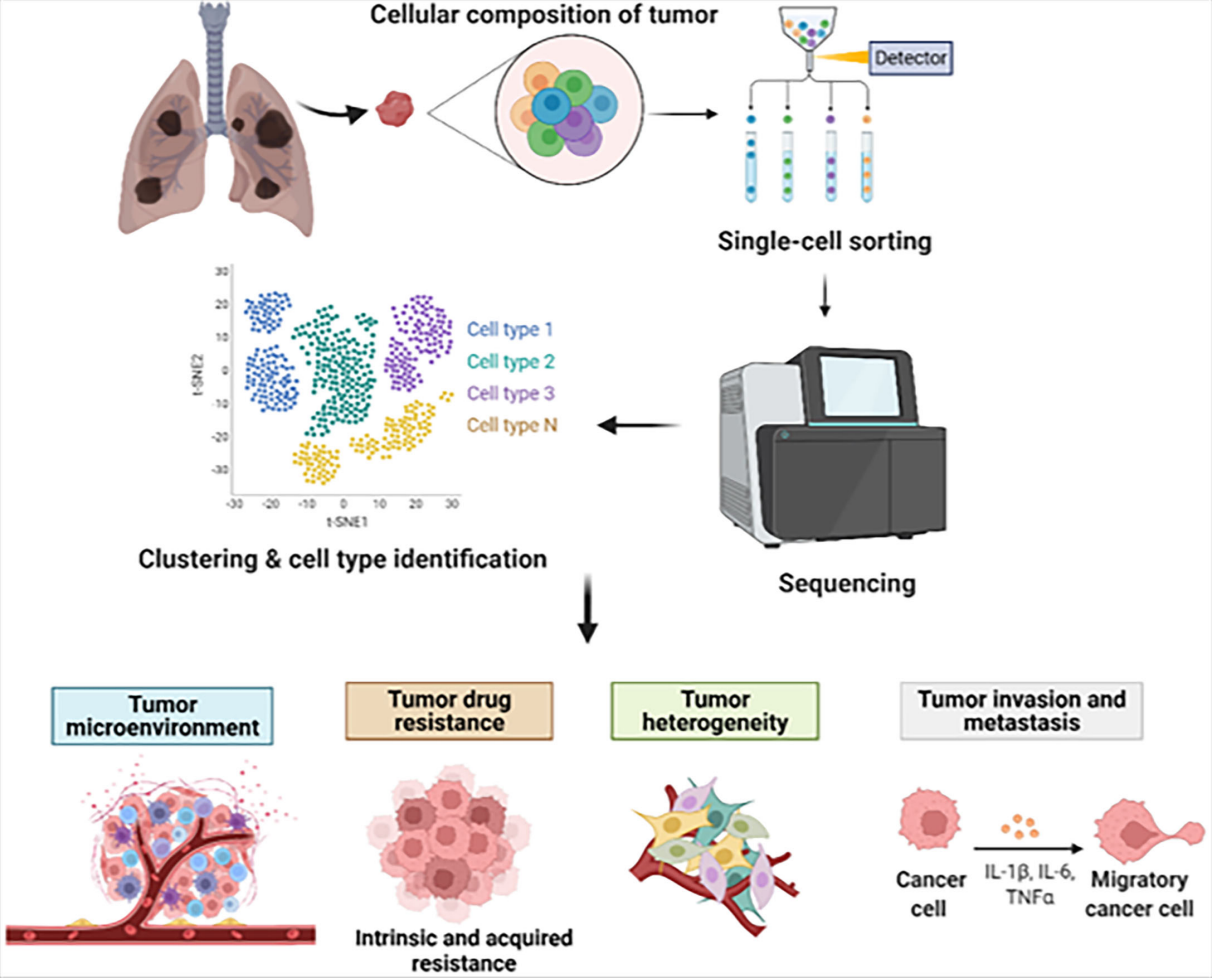
Decoding lung cancer at single-cell level.

In this minireview, we summarized recent applications of single-cell analysis in studying pathogenesis of lung cancer, potential contribution for investigating novel clinical treatments, identification of biomarkers, and delineation the resistant mechanism in lung cancer (**Figure 1**). Since single-cell analysis can accurately capture very rich information of tumor heterogeneity and TME at multi-levels, in-depth mining of lung cancer single-cell data can achieve better patient stratification and explore more new therapeutic targets for precision medicine.

In future, integration of single-cell analysis with clinical therapies shall be a promising strategy to accelerate the development of personalized medicine and avoid the occurrence of drug resistance. Furthermore, coupling single-cell sequencing with spatial transcriptomics could be an efficient way to study the progression of lung cancer (36, 37).

However, there are still challenges which limit the use of single-cell analysis. For example, optimalisation is always needed to avoid destruction/loss of valid cells, low coverage, and biased sequencing results. Also, for single cell proteomics, it is hard to accurately quantify the level of proteins from single cells. Finally, how the analyzed cells can be typically reflect the temporal/spatial specificity of tumor tissue in patients is often lack of confidence. Hence, more advanced single cell analysis techniques are required to further optimize personalized clinical strategies to treat lung cancer and other human diseases.

## Author Contributions

Both authors contributed to the article and approved the submitted version.

## Funding

This work was funded by Macau Science and Technology Development Fund project (Grant no. 0003/2018/A1 and 0058/2020/A2) and Macau University of Science and Technology foundation (Grant no. FRG-20-003-SKL) granted to Dr. Xing-Xing Fan. Dr. Neher's Biophysics Laboratory for Innovative Drug Discovery (Grant no. 001/2020/ALC). Qiang Wu is supported by Macau University of Science and Technology foundation (0072/2019/A2).

## Conflict of Interest

The authors declare that the research was conducted in the absence of any commercial or financial relationships that could be construed as a potential conflict of interest.

## Publisher’s Note

All claims expressed in this article are solely those of the authors and do not necessarily represent those of their affiliated organizations, or those of the publisher, the editors and the reviewers. Any product that may be evaluated in this article, or claim that may be made by its manufacturer, is not guaranteed or endorsed by the publisher.
